# Neurologic immune-related adverse events associated with adjuvant ipilimumab: report of two cases

**DOI:** 10.1186/s40425-018-0393-z

**Published:** 2018-08-31

**Authors:** Christine A. Garcia, Alex El-Ali, Tanya J. Rath, Lydia C. Contis, Vikram Gorantla, Jan Drappatz, Diwakar Davar

**Affiliations:** 1 Hillman Cancer Center, University of Pittsburgh Medical Center, 5150 Centre Ave, Pittsburgh, PA 15232 USA; 2 Department of Radiology, University of Pittsburgh Medical Center, PUH Suite E204, 200 Lothrop Street, Pittsburgh, PA 15213 USA; 3 Division of Hematopathology, Department of Radiology, University of Pittsburgh Medical Center, PUH Suite E204, 200 Lothrop Street, Pittsburgh, PA 15213 USA

**Keywords:** Cancer, Melanoma, Immunotherapy, Ipilimumab, Infliximab, Immune-related adverse events, CTLA-4, TNF-α, Neurotoxicity, Autoimmune, Guillain Barre syndrome, Meningoencephalomyelitis

## Abstract

**Background:**

PD-1 and CTLA-4 inhibitors are associated with several adverse events including a spectrum of immune-related adverse effects (irAEs). Neurologic irAEs are uncommon occurrences with varied presentations. We describe two separate cases of ipilimumab associated meningoencephalomyelitis and demyelinating polyneuropathy with unusual presentations.

**Case presentation:**

Two melanoma patients were treated with ipilimumab in the adjuvant setting. The first patient developed a meningoencephalitis following 3 doses of ipilimumab. MRI imaging of the brain confirmed leptomeningeal enhancement although cerebrospinal fluid (CSF) analyses were negative for malignant cells consistent with meningoencephalomyelitis. Although she initially improved following treatment with steroids and intravenous immunoglobulin, she subsequently relapsed. She was successfully treated with infliximab and made a complete neurological recovery. A second patient developed progressive lower extremity weakness following two doses of ipilimumab. MRI imaging of the spine confirmed diffuse nerve root enhancement consistent with acute inflammatory demyelinating polyneuropathy (AIDP). He was treated with high dose steroids with resolution of neurological symptoms. Both patients remain disease free.

**Conclusions:**

Neurological irAEs are uncommon adverse events in the context of CTLA-4 and/or PD-1 inhibitor therapy. Care must be taken to distinguish these from leptomeningeal disease. Early recognition of neurological irAEs is critical for the initiation of specific anti-inflammatory agents to prevent and potentially reverse neurological sequelae.

## Background

Cytotoxic T lymphocyte antigen-4 (CTLA-4) and CD28 are homologues with diametric effects on T cell activation. While the B7/CD28 interaction provides the second “costimulatory” signal required for T cell activation; by cross-linking CD28 and T-cell receptor (TCR) CTLA-4 strongly inhibits T cell activation [1]. Consequently, inhibition of CTLA-4 by monoclonal antibodies such as ipilimumab and tremelimumab promotes anti-tumor immunity [2]. Ipilimumab is a fully humanized IgG1 monoclonal antibody that inhibits the CD28/CTLA-4 interaction, thereby promoting T cell activation [3] and resulting in anti-tumor immunity [4]. Ipilimumab therapy improves survival in metastatic melanoma and results in durable remissions with 3-year survival rates of 20% and 26% in treated and treatment-naïve melanoma patients respectively [5–7].

Ipilimumab is associated with several adverse events including a spectrum of immune-related adverse effects (irAEs) that include enterocolitis, pneumonitis, hepatitis, dermatitis, hypophysitis and nephritis [8]. Incidence of irAEs in phase II and III trials differ widely: dermatitis (47–68%), enterocolitis (31–46%), hepatitis (3–9%), hypophysitis (4–6%), pancreatitis (1.5%), uveitis (1%), lymphadenopathy (1%) and neurologic events (0.1%) [9]. The incidence of neurologic irAEs in ipilimumab phase II and III trials was 0.1% with no grade 3–4 events [10], although cases of nerve palsies, demyelination, limbic encephalitis, Guillain-Barre Syndrome (GBS) and myasthenia gravis have been reported [9]. The etiology of ipilimumab-induced irAEs is unclear; possible explanations include the loss of peripheral tolerance mediated by CTLA-4 and ectopic expression of CTLA-4 at least in the setting of hypophysitis [11].

Well-established algorithms for the management of neurologic irAEs associated with ipilimumab and nivolumab have been published [12]. Management guidelines encourage early use of high-dose steroids and discontinuation of immuno-oncologic agents for grade 2 events; while recommending specialist neurologic input and intravenous immunoglobulin (IVIG) for grade 3–4 events [13]. Unlike in colitis wherein the role of TNF-α inhibitors is clear [14, 15], the role of these agents in the management of grade 3/4 neurologic irAEs refractory to steroids and IVIG is unknown.

In this report, we describe two patients with melanoma who received ipilimumab in the adjuvant setting and developed ipilimumab-related neurologic irAEs. The first patient developed a meningoencephalomyelitis that relapsed despite high dose steroids and IVIG, but subsequently responded to a course of infliximab. The second patient developed an acute inflammatory demyelinating polyneuropathy (AIDP) that responded to high dose steroids. In this report, we highlight the importance of symptom recognition and appropriate diagnostic evaluation to early diagnosis of unusual neurologic irAEs permitting institution of appropriate immunosuppressive therapy.

## Case presentation

### Patient 1

**Fig. 1:**
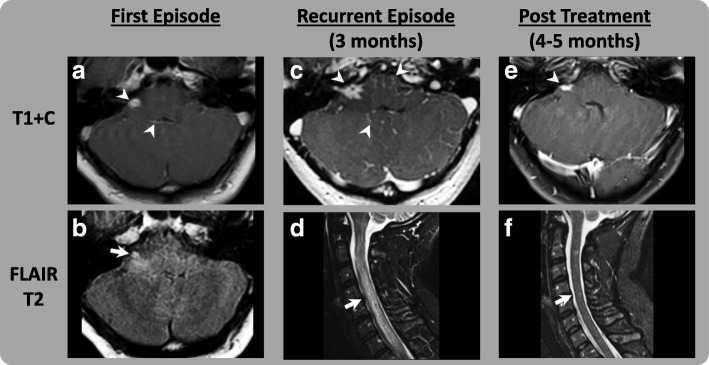
Gadolinium Enhanced MRI Brain and Spine, Images of Patient 1. First Episode (**a** and **b**): Abnormal nodular leptomeningeal enhancement, most notably at the R-cerebellar pontine angle (CPA) and 4^th^ ventricle (arrowhead). Lack of FLAIR suppression at the basal cisterns is also most prominent at the R-CPA. There is associated parenchymal edema that extends into the right brachium pontis (arrow). Recurrent Episode (**c** and **d**): Worsening leptomeningeal enhancement, prominently involving the R-internal auditory canal (arrowhead). Holocord T2 signal hyperintensity consistent with edema resulting in complete effacement of the CSF space (arrow). Post-treatment (**e** and **f**): Mild residual abnormal nodular enhancement at the right IAC (arrowhead) and near complete normalization of the cord diameter. There is only trace residual abnormal signal (arrow)

A 39-year-old Caucasian female was diagnosed with nodular melanoma of the right upper back following a biopsy. Wide local excision (WLE) and right axillary sentinel lymph node (SLN) biopsy were performed; although WLE was negative, SLN was positive for multiple foci of residual melanoma in two LN. Completion lymph node dissection (CLND) was negative for any involvement of 19 LN. She was staged with IIIA (T3aN2aMx) disease and offered adjuvant therapy with ipilimumab based on the EORTC 18071 data [16]. Between January 4, 2017 and February 24, 2017, she received 3 doses of ipilimumab 10 mg/kg IV. Prior to fourth induction dose of ipilimumab, she developed flu-like symptoms and persistent headaches although no neurologic deficits were noted. Magnetic resonance imaging (MRI) of the brain and spine showed mild pituitary enlargement and leptomeningeal enhancement with a nodular focus of enhancement in the right internal auditory canal (Fig. 1a and b). On lumbar puncture, opening pressures were elevated (> 30 mm water) while cerebrospinal fluid (CSF) examination was negative for any malignant cells but showed lymphocytic pleocytosis. Extensive testing excluded infectious and autoimmune etiologies; and other laboratory tests were consistent with central hypothyroidism and adrenal insufficiency. She was diagnosed with ipilimumab-related meningoencephalomyelitis, and treated with methylprednisolone 1.0 mg/kg daily with rapid improvement and discharged on an oral prednisone taper. As an outpatient, prednisone was gradually tapered over an eight-week period till she was on physiologic doses of hydrocortisone. She was also treated with levothyroxine 100 mcg daily for ipilimumab-related hypothyroidism. Ipilimumab adjuvant therapy was permanently discontinued.

Approximately 3 months following initial admission, she developed lower extremity weakness and fecal and urinary incontinence. MRI imaging of the brain and spine showed recurrent leptomeningeal and cranial nerve enhancement; diffuse lack of FLAIR suppression of CSF signal; and expansion of the cervical cord and T2 hyper-intense cord signal alteration with abnormal patchy intramedullary enhancement (Fig. 1c and d). CSF studies documented recurrent lymphocytic pleocytosis while serological studies were negative for autoimmune etiologies including paraneoplastic syndromes. Her CSF protein was elevated to 120 mg/dL (range 15–45 mg/dL). Imaging was negative for any evidence of recurrent malignancy. Per American Society of Clinical Oncology (ASCO) clinical practice guidelines, IVIG 1 g/kg and methylprednisolone 1.0 mg/kg IV daily were administered for 5 days albeit with scant improvement. In the setting of IVIG/steroid refractory ipilimumab-related meningoencephalomyelitis, a trial of infliximab was offered. Infliximab 5 mg/kg IV was administered while prednisone 1 mg/kg daily was continued with improvement in neurologic function [17]. Two days after first dose of infliximab, she was able to ambulate with support although she continued to have urinary retention. Subsequently, she was discharged for spinal cord rehabilitation. Infliximab 5 mg/kg was continued for a further two doses at two and 4 weeks following initial administration. Prednisone was continued at 60 mg daily for 2 months then tapered gradually by 10 mg every 2 weeks over a 3-month period after which she was transitioned to physiologic replacement doses of hydrocortisone on which she remains indefinitely. Her headaches and neurologic symptoms have improved substantially with near complete neurologic recovery including continence except mild spasticity in lower extremities. Repeat MRI imaging of the brain and spine documented progressively decreased leptomeningeal enhancement and eventual normalization of T2 signal (Fig. 1e and f). Restaging scans remain negative for recurrent melanoma.

### Patient 2

**Fig. 2:**
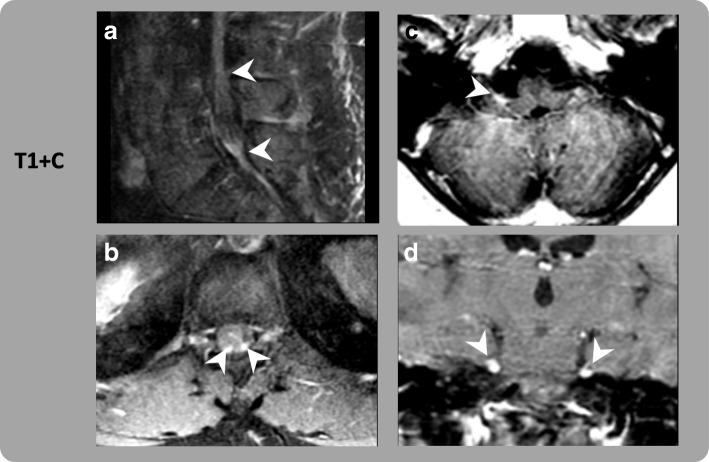
Gadolinium Enhanced MRI Brain and Spine, Images of Patient 2. Post-contrast MR images demonstrate abnormal leptomeningeal enhancement throughout the CNS. (**a**) abnormal smooth thickening and enhancement of the cauda equina and nerve roots (arrowhead). (**b**) abnormal smooth thickening of the nerve roots at the thoracolumbar junction (arrowhead). (**c**) abnormal enhancement of the 7^th^ cranial nerve (arrowhead). (**d**) abnormal enhancement of both 5^th^ cranial nerves seen in a coronal plane (arrowhead)

A 55 year-old male was diagnosed with stage IIIB (T4bN1aMx) superficial spreading melanoma of the right distal thigh after initial surgery, right inguinal SLN biopsy and right inguinal CLND. He was treated with ipilimumab 10 mg/kg IV every 3 weeks and received 2 doses. Following second dose, he developed parasthesias in distal lower extremities bilaterally that subsequently ascended to involve proximal lower extremities, then upper extremities along with weakness and loss of deep tendon reflexes. No bladder or bowel incontinence and/or difficulties with speech were observed. MRI brain and whole spine revealed abnormal enhancement involving the bilateral 5th, 7th and 8th cranial nerves, cauda equina nerve roots as well as the conus surface and peripheral nerves at the thoracolumbar junction **(**Fig. 2a and d)**.** CSF studies were negative for malignant cells but showed lymphocytic pleocytosis. CSF protein was elevated to 175 mg/dL (range 15–45 mg/dL). An extensive evaluation for autoimmune and paraneoplastic etiologies was negative although laboratory studies revealed concomitant adrenal insufficiency. Electromyography showed electrophysiologic evidence consistent with a generalized, sensory and motor, length-dependent, predominantly axonal, peripheral polyneuropathy without definite electrophysiologic evidence of a presynaptic neuromuscular junction transmission disorder or proximal myopathy. He was diagnosed with ipilimumab-related acute inflammatory demyelinating polyneuropathy (AIDP).

Methylprednisolone 1.5 mg/kg twice daily was initiated with rapid improvement in motor symptoms in hands and lower extremities after 2 days. Steroids were tapered slowly from prednisone 1 mg/kg by decreasing by 10 mg weekly over a 12-week period with gradual recovery. Towards the end of the taper, prednisone was transitioned to physiologic doses of hydrocortisone to treat ipilimumab-related adrenal insufficiency. Although weakness resolved completely, he continued to have minimal parasthesias in upper extremities and lower extremities in a glove-and-stocking distribution. Approximately 4 months after initial ipilimumab therapy, he developed hypothyroidism for which he was treated with levothyroxine 125mcg daily. Systemic imaging studies remain negative for recurrent disease.

## Discussion

Ipilimumab-associated neurological irAEs span diverse entities including severe meningo-radiculo-neuritis [18], reversible splenial lesions [19, 20], hypophysitis [21], meningitis [21], acute cerebellitis [22], Guillain-Barre syndrome [21, 23], myasthenia gravis [24–27], bilateral phrenic nerve paralysis [28], Bell’s palsy [28], chronic inflammatory demyelinating polyneuropathy (CIDP) [29], transverse myelitis [29], encephalitis [30], necrotic myelopathy [31], and partial motor convulsive status (epilepsia partialis continua) [32]. Herein, we report two cases of ipilimumab-associated neurological irAEs manifesting as meningoencephalomyelitis and AIDP respectively. Although ipilimumab-related meningoencephalomyelitis and AIDP have both been reported previously, this report describes two patients with diverse manifestations including a relapse following initial immunosuppression requiring infliximab for resolution. Both patients also developed autoimmune thyroiditis and adrenal insufficiency in addition to neurological irAEs, suggesting that several immune-related adverse events including neurological manifestations can cluster. A review of previously published reports of ipiliumumab-associated neurological irAEs suggests that neurological irAEs occur both in isolation and with other irAEs with no consistent pattern linking the two (Table 1**).**

**Table 1:** Case Reports/Series of Ipilimumab-Associated Neurological irAEs

Case reports/series	Neurological manifestations	Presence of additional irAEs
Wilgenhof S, et al. (2011) [23]	Guillain-Barré Syndrome	None reported
Bompaire F, et al. (2012) [18]	Meningo-radiculo-neuritis	None reported
Bot I, et al. (2013) [21] (*n* = 3)	Hypophysitis Meningitis Guillain-Barré syndrome	
Liao B. et al. (2014) [21] (*n* = 3)	CIDP Transverse myelitis Myasthenia gravis-type syndrome	None reported
Johnson DB, et al. (2015) [24]	Myasthenia gravis	Thyroiditis Dermatitis
Thaipisuttikul I, et al.(2015) [35]	Peripheral neuropathy	None reported
Abdallah AO, et al. (2016) [31]	Necrotic myelopathy	None reported
Chen JH, et al. (2017) [27]	Myasthenia gravis	Myositis Polyneuropathy Hepatitis
Derle E, et al. (2018) [25]	Myasthenia gravis	None reported
Montes V, et al. (2018) [26]	Myasthenia gravis	None reported
Naito T, et al. (2018) [22]	Acute cerebellitis	None reported
Ruff MW, et al. (2018) [28] (*n* = 2)	Bilateral phrenic nerve paralysis Bell’s palsy	Pan-hypopituitarism

The tumor necrosis factor alpha (TNF-α) inhibitor infliximab has a clear role in the management of steroid-refractory immune-related colitis [15, 33, 34], however its role in the treatment of neurologic irAE is unknown. In a prior report, infliximab was administered to treat severe ipilimumab-induced peripheral neuropathy that developed after two doses of adjuvant ipilimumab administered in the setting of stage III melanoma [35]. However, irAE was refractory to steroids, IVIG and infliximab and ultimately required tacrolimus for resolution. In another case report, infliximab (5 mg/kg IV) was given monthly for ipilimumab-induced necrotic myelopathy after lack of response to daily prednisone (1 mg/kg) for 2 weeks [31]. Four weeks after infliximab therapy, she is still wheelchair bound with painful paresthesia.

Exact mechanisms underlying CTLA-4 inhibitor toxicity remain unclear, although multiple hypotheses including loss of peripheral tolerogenic mechanisms via T-regulatory cell depletion [36, 37] and atypical expression of CTLA-4 on non-immune cells [11] have been advanced. Given links between CTLA-4 gene polymorphisms (in particular CTLA-4 59A > G variant) and incidence of autoimmune diseases, it is possible that CTLA-4 gene polymorphisms may mediate a certain fraction of irAEs related to CTLA-4 inhibition. Expression of certain major histocompatibility complex (MHC) or human leucocyte antigen (HLA) class-I and -II proteins (in particular HLA-DR) is associated with response to PD-1 blockade [38, 39]. However, whether certain MHC haplotypes have an increased risk of irAEs when treated with PD-1 and/or CTLA-4 inhibitor therapy is less well understood. The link between certain autoimmune diseases and particular HLA haplotypes is well known including DQB1 in GBS [39, 40]. Interestingly, both patients were haplo-identical at the HLA-DRB1*04, HLA-DRB4*01 and HLA-DQB1*03 alleles (Table 2). In the absence of larger studies with longer term follow up, the significance of this association is unknown.

**Table 2:** HLA Typing Results from Patients 1 and 2

HLA Class I	A	B	Bw	C	Cw			
Patient 1	24:XX	07:XX	06:XX	04:XX	04:XX			
	29:XX	35:XX	06:XX	07:XX	07:XX			
Patient 2	11:01	27:05	04:XX	01:02				
	24:02	35:01	06:XX	04:01				
HLA Class II	DRB1	DRB3	DRB4	DRB5	DQB1	DQA1	DPB1	DRw
Patient 1	04:XX		01:XX	01:XX	03:XX			53:XX
	15:XX				05:XX			51:XX
Patient 2	01:01		01:03		03:02	01:01	03:01	
	04:04		53:XX		05:01	03:01	14:01	

The incidence of ipilimumab-related irAEs is clearly dose-related with a significantly greater incidence of irAEs at the 10 mg/kg dose compared to the 3 mg/kg dose [5, 6]. However, the irAE incidence at the 10 mg/kg dose is similar in both adjuvant and metastatic disease settings [6, 16]. This data suggests that irAE occurrence depends several upon host factors but may be agnostic of the disease burden. Recent reports have implicated interactions between the intestinal microbiome and T regulatory cells in mediating some aspect of ipilimumab related colitis but whether this extends to ipilimumab related neurologic irAEs is as yet unknown [41–43].

## Conclusions

In this report, we describe two patients with melanoma who received ipilimumab in the adjuvant setting and developed distinct ipilimumab-related neurologic irAEs. Early recognition and management of neurological irAEs is essential for maximizing clinical recovery and minimizing effect of drug-related toxicity. Although irAEs can occur at any stage of treatment, presentations within 4 months of treatment initiation is typical [44] as was seen in both these patients. Management of suspected neurological irAEs should incorporate early input of subspecialty neurologists attuned to fully characterize the neurological perturbation and to exclude leptomeningeal and/or paraneoplastic manifestations of cancer particularly in certain malignancies (melanoma, non-small cell lung cancer, etc.). Early initiation of immunosuppression with corticosteroids for grade 2 or greater events is critical; and steroids should be tapered slowly to avoid flares. The successful use of infliximab to treat a severe steroid/IVIG-refractory neurologic irAE reported herein suggests that clinicians can consider the role of alternative immunosuppressive agents (mycophenolate, infliximab, etc.) to treat steroid-refractory neurological irAEs.

## Abbreviations

AIDP: Acute inflammatory demyelinating polyneuropathy
CLND: Complete lymph node dissection
CSF: Cerebral spinal fluid
CTLA: Cytotoxic T-lymphocyte antigen-4
EORTC: European Organisation for Research and Treatment of Cancer
GBS: Guillain-Barre Syndrome
irAEs: Immune-related adverse effects
IV: intravenous
MRI: Magnetic resonance imaging
PD-1: Programmed cell death protein 1
SLN: Sentinel lymph node
WLE: Wide local excision
